# Clustering of Pattern Recognition Receptors for Fungal Detection

**DOI:** 10.1371/journal.ppat.1003873

**Published:** 2014-02-20

**Authors:** Makoto Inoue, Mari L. Shinohara

**Affiliations:** 1 Department of Immunology, Duke University School of Medicine, Durham, North Carolina, United States of America; 2 Department of Molecular Genetics and Microbiology, Duke University School of Medicine, Durham, North Carolina, United States of America; The University of North Carolina at Chapel Hill, United States of America

## Introduction

The innate immune system is the first line of defense against invading pathogens. Innate cells recognize microbes via pattern recognition receptors (PRRs), such as Toll-like receptors (TLRs) and C-type lectin receptors (CLRs); initiate innate immune responses; and eventually trigger adaptive immunity. Association of heterologous PRRs synergistically enhances their signal intensity. Such PRR cluster formation is essential for fungal detection in generating antifungal immunity. In this review, PRR cluster formation to detect fungi and to initiate innate immune responses is discussed.

## PRRs Involved in Fungal Detection

Upon fungal infection, host innate immune cells, such as macrophages, neutrophils, and dendritic cells (DCs), are the first cell types to detect the infection. During the early stages of infection, before T cells are activated, the host innate immune system initiates antifungal responses (i.e., phagocytosis; production of reactive oxygen species [ROS], cytokines, and chemokines) as sentinels. Antigen-presenting myeloid cells, such as DCs and macrophages, then activate T cells by presenting foreign antigens to trigger adaptive immune responses, along with secretion of cytokines and chemokines to instruct naïve T cells to polarize “proper” T cell subsets.

Several TLRs (including TLR1/2, TLR2/6, TLR4, and TLR9) and CLRs (including dectin-1, dectin-2, MMR [Macrophage Mannose Receptor, CD206], and DC-SIGN [Dendritic Cell-Specific Intercellular adhesion molecule-3-Grabbing Non-integrin, CD209]) play roles as fungal sensors. Among these, TLR2 and dectin-1 are the best-characterized PRRs in detecting fungi such as *Pneumocystis* [1] and *Candida albicans* [2], [3]. TLR2 heterodimerizes with either TLR1 or TLR6 and recognizes triacylated and diacylated lipoprotein as TLR1/2 and TLR2/6, respectively. TLR4 and TLR9 recognize O-linked mannosyl residues on fungal walls and fungal DNA, respectively. TLR signaling pathways activate NFκB, which is a key transcription factor for the production of cytokines and chemokines.

Dectin-1, expressed by macrophages, neutrophils, and DCs, recognizes β-glucans in the fungal cell walls [3]. Stimulation of dectin-1 triggers phagocytosis of fungi, ROS generation, and production of cytokines and chemokines by host innate immune cells. Dectin-1 is a critical PRR in host defense against fungal infection, since TLR stimulation alone is not sufficient to induce ROS [4]. Dectin-1 signaling activates Syk (Spleen Tyrosine Kinase), and thus leads to subsequent activation of NFκB and NFAT (Nuclear Factor of Activated T-cells). NFκB is activated by a signaling adaptor complex consisting of CARD9 (CAspase Recruitment Domain-containing protein-9), BCL10 (B Cell Lymphoma/leukemia-10), and MALT1 (Mucosa-Associated Lymphoid Tissue lymphoma translocation protein-1) [5]. On the other hand, NFAT activation by dectin-1 requires PLCγ2 (Phospholipase C-gamma 2) signaling [6]. Dectin-1–mediated Syk signaling also activates the NLRP3 (NOD-Like Receptor family, Pyrin domain containing 3) inflammasome, which mediates IL-1β and IL-18 maturation, and it plays an important role in the host defense against *C. albicans* [7]. Other CLRs, dectin-2, dectin-3, MMR, and DC-SIGN, recognize mannan [8]. Recent study has shown that dectin-2 and dectin-3 form a heterodimer, which binds to α-mannan more effectively and elicits more potent inflammatory responses than does a dectin-2 homodimer [8]. Chitin recognition is considered to be a size-dependent process and involves a combination of dectin-1 and MMR, with or without TLR2 [9].

## Synergy of PRRs

It is known that association of heterologous PRRs synergistically enhances their signal intensity. TLR2, dectin-1, and MMR play a critical role in the detection and clearance of *Pneumocystis* [1], [10]. In particular, TLR2 and dectin-1 physically associate each other and synergize to augment antifungal responses [4], [10]–[12]. Co-localization of TLR2 and dectin-1 in the phagocytic cap engulfing zymosan on the macrophage membrane was also reported [10], [13]. Zymosan does not activate NFκB when cells express either TLR2 or dectin-1 alone; however, co-expression of TLR2 and dectin-1 allows zymosan to activate NFκB [4], suggesting the importance of PRR synergy. Synergy of decin-1 and TLR2 was also demonstrated in human monocyte-derived macrophages via increased TNFα production with curdlan (dectin-1 ligand) and Pam3Cys (TLR2 ligand) co-stimulation [12]. Further, crosstalk between dectin-1 and TLR2 signaling pathways has been suggested from the failure to elicit collaborative receptor responses by the lack of a signaling molecule, Syk or MyD88, which are downstream of dectin-1 and TLR2, respectively [11]. In addition to TLR2, pathways of TLR4, TLR5, TLR7, and TLR9 appear to collaborate with the dectin-1/Syk signaling pathway based on the synergistic enhancement of TNF production [11], [12]. Finally, it has been reported that DC-SIGN synergizes TLR signaling through RAF1, a serine/threonine kinase, which induces phosphorylation of the p65 subunit of NFκB, followed by prolonged and enhanced transcription of *Il10* by NFκB activation [14]. A sizeable number of studies thus suggest the existence of synergistic collaboration, particularly between TLR and CLR signaling pathways.

## PRR Clustering in Fungal Detection

**Figure 1:**
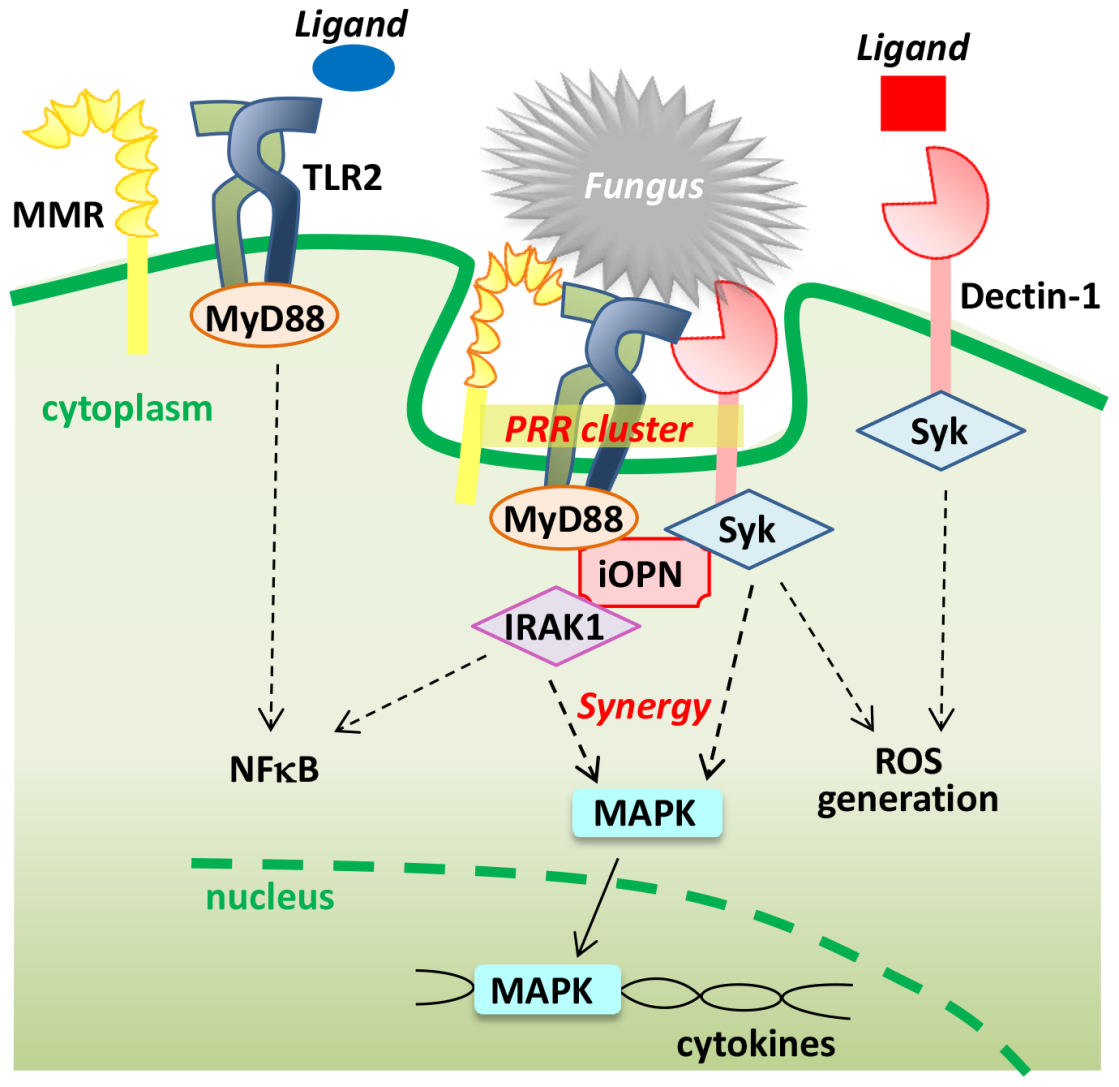
A model example of a PRR cluster formation for fungal detection. Soluble ligands do not make a PRR cluster, but a fungal spore or a particulate ligand attracts PRRs in a lipid raft on the host cell surface. In this figure, heterologous PRRs, consisting of dectin-1, TLR2, and MMR, form a PRR cluster.

In order to achieve synergy between heterologous PRR signaling pathways, it is considered crucial to have PRRs in physical proximity to one another. Clustering PRRs on a myeloid cell surface is required for the optimum detection of fungi [10], [15]. TLR2 and dectin-1 are recruited to lipid rafts, plasma membrane microdomains rich in cholesterol and sphingolipids, after stimulation with their ligands; signals from the receptors are synergized to enhance the downstream responses [10], [16]. Disruption of lipid rafts causes decreased cytokine expression, phagocytosis, Ca^2+^ influx, and phosphorylation of both Syk and ERK (Extracellular signal Regulated Kinases) [16], suggesting the critical role of rafts in PRR signaling. Importance of receptor cluster formation in antifungal immunity was demonstrated by comparing soluble and particulate β-glucans immobilized on latex beads [15]. Treating bone marrow–derived macrophages with particulate β-glucans induced dectin-1 signaling but soluble β-glucans did not; the former was done through the mechanism of excluding regulatory tyrosine phosphatases CD45 and CD148 from synapse-like structures [15]. We speculate that the physical crosslink of PRRs by particulate ligands may also be critical for triggering strong receptor signaling [10]. The PRR cluster was termed “phagocytic synapse” [15], which provides a platform for fungal detection and phagocytosis (Figure 1). Such PRR clustering is reminiscent of the supramolecular activation cluster (SMAC) formed on T cell surfaces in the immunological synapse; although, PRR clusters do not appear to be as distinct and intricate as SMAC. The biology and precise mechanism of PRR clustering are clearly arenas for further research.

We reported that PRR cluster formation of TLR2 and dectin-1 is essential for antifungal innate immunity against *Pneumocystis* [10]. In the signaling pathway downstream of the TLR2/dectin-1 cluster, intracellular osteopontin (iOPN) [17] plays a critical role (Figure 1). Upon detection of *Pneumocystis*, iOPN was essential for the clustering of TLR2, dectin-1, and MMRs in macrophages [10]. iOPN simultaneously associates with IRAK1 and Syk, which are signaling molecules downstream of TLR2 and dectin-1, respectively, while enhancing MAPK (Mitogen-Activated Protein Kinases) activation [10]. OPN-deficient macrophages have shown attenuated phagocytosis of *Pneumocystis* and ROS generation, as well as cytokines production, suggesting that iOPN is essential for antifungal effects through PRR cluster formation. In fact, OPN-deficient immunocompromised mice are extremely susceptible to opportunistic infection by *Pneumocystis* [10]. iOPN is known to co-localize with actin and induces cytoskeletal rearrangement [18], [19]. It is also known that the actin cytoskeleton plays an important role in the dynamics of the clusters with transmembrane receptors [20]. Therefore, evaluating the role of iOPN in the PRR clustering in the context of cytoskeletal rearrangement upon fungal detection may be of interest.

## Question to Be Asked

Depending on the fungal species to be detected, macrophages appear to form clusters comprised of a unique combination of PRRs to optimize detection of fungi and to create synergy among multiple PRRs. However, precisely how PRRs are recruited to the lipid rafts and how the synergistic signal transduction pathways crosstalk are not clear. Relative to viruses and bacteria, sizes of fungi are, in general, significantly larger. It is therefore logical for host cells to form PRR clusters in an effort to maximize the interaction between PRRs and the fungal surface. In this regard, we suggest that the mechanism of PRR cluster formation in host cells during fungal detection is an issue critical to our further understanding of detection of fungi by host cells.

## References

[1] Zhang C, Wang SH, Lasbury ME, Tschang D, Liao CP, et al (2006) Toll-like receptor 2 mediates alveolar macrophage response to Pneumocystis murina. Infect Immun 74: 1857–1864. 16495560 10.1128/IAI.74.3.1857-1864.2006 PMC1418649

[2] Villamon E, Gozalbo D, Roig P, O'Connor JE, Fradelizi D, et al (2004) Toll-like receptor-2 is essential in murine defenses against Candida albicans infections. Microbes Infect 6: 1–7. 14738887 10.1016/j.micinf.2003.09.020

[3] Taylor PR, Tsoni SV, Willment JA, Dennehy KM, Rosas M, et al (2007) Dectin-1 is required for beta-glucan recognition and control of fungal infection. Nat Immunol 8: 31–38. 17159984 10.1038/ni1408 PMC1888731

[4] Gantner BN, Simmons RM, Canavera SJ, Akira S, Underhill DM (2003) Collaborative induction of inflammatory responses by dectin-1 and Toll-like receptor 2. J Exp Med 197: 1107–1117. 12719479 10.1084/jem.20021787 PMC2193968

[5] Gross O, Gewies A, Finger K, Schafer M, Sparwasser T, et al (2006) Card9 controls a non-TLR signalling pathway for innate anti-fungal immunity. Nature 442: 651–656. 16862125 10.1038/nature04926

[6] Xu S, Huo J, Lee KG, Kurosaki T, Lam KP (2009) Phospholipase Cgamma2 is critical for Dectin-1-mediated Ca2+ flux and cytokine production in dendritic cells. J Biol Chem 284: 7038–7046. 19136564 10.1074/jbc.M806650200 PMC2652331

[7] Gross O, Poeck H, Bscheider M, Dostert C, Hannesschlager N, et al (2009) Syk kinase signalling couples to the Nlrp3 inflammasome for anti-fungal host defence. Nature 459: 433–436. 19339971 10.1038/nature07965

[8] Zhu LL, Zhao XQ, Jiang C, You Y, Chen XP, et al (2013) C-type lectin receptors Dectin-3 and Dectin-2 form a heterodimeric pattern-recognition receptor for host defense against fungal infection. Immunity 39: 324–334. 23911656 10.1016/j.immuni.2013.05.017

[9] Mullin NP, Hitchen PG, Taylor ME (1997) Mechanism of Ca2+ and monosaccharide binding to a C-type carbohydrate-recognition domain of the macrophage mannose receptor. J Biol Chem 272: 5668–5681. 9038177 10.1074/jbc.272.9.5668

[10] Inoue M, Moriwaki Y, Arikawa T, Chen YH, Oh YJ, et al (2011) Cutting edge: critical role of intracellular osteopontin in antifungal innate immune responses. J Immunol 186: 19–23. 21135164 10.4049/jimmunol.1002735 PMC3538145

[11] Dennehy KM, Ferwerda G, Faro-Trindade I, Pyz E, Willment JA, et al (2008) Syk kinase is required for collaborative cytokine production induced through Dectin-1 and Toll-like receptors. Eur J Immunol 38: 500–506. 18200499 10.1002/eji.200737741 PMC2430329

[12] Ferwerda G, Meyer-Wentrup F, Kullberg BJ, Netea MG, Adema GJ (2008) Dectin-1 synergizes with TLR2 and TLR4 for cytokine production in human primary monocytes and macrophages. Cell Microbiol 10: 2058–2066. 18549457 10.1111/j.1462-5822.2008.01188.x

[13] Brown GD, Herre J, Williams DL, Willment JA, Marshall AS, et al (2003) Dectin-1 mediates the biological effects of beta-glucans. J Exp Med 197: 1119–1124. 12719478 10.1084/jem.20021890 PMC2193964

[14] Gringhuis SI, den Dunnen J, Litjens M, van Het Hof B, van Kooyk Y, et al (2007) C-type lectin DC-SIGN modulates Toll-like receptor signaling via Raf-1 kinase-dependent acetylation of transcription factor NF-kappaB. Immunity 26: 605–616. 17462920 10.1016/j.immuni.2007.03.012

[15] Goodridge HS, Reyes CN, Becker CA, Katsumoto TR, Ma J, et al (2011) Activation of the innate immune receptor Dectin-1 upon formation of a ‘phagocytic synapse’. Nature 472: 471–475. 21525931 10.1038/nature10071 PMC3084546

[16] Xu S, Huo J, Gunawan M, Su IH, Lam KP (2009) Activated dectin-1 localizes to lipid raft microdomains for signaling and activation of phagocytosis and cytokine production in dendritic cells. J Biol Chem 284: 22005–22011. 19525229 10.1074/jbc.M109.009076 PMC2755924

[17] Inoue M, Shinohara ML (2011) Intracellular osteopontin (iOPN) and immunity. Immunol Res 49: 160–172. 21136203 10.1007/s12026-010-8179-5 PMC3509172

[18] Shinohara ML, Kim HJ, Kim JH, Garcia VA, Cantor H (2008) Alternative translation of osteopontin generates intracellular and secreted isoforms that mediate distinct biological activities in dendritic cells. Proc Natl Acad Sci U S A 105: 7235–7239. 18480255 10.1073/pnas.0802301105 PMC2438233

[19] Suzuki K, Takeyama S, Kikuchi T, Yamada S, Sodek J, et al (2005) Osteoclast responses to lipopolysaccharide, parathyroid hormone and bisphosphonates in neonatal murine calvaria analyzed by laser scanning confocal microscopy. J Histochem Cytochem 53: 1525–1537. 16087705 10.1369/jhc.5A6630.2005 PMC3957542

[20] Gonnord P, Blouin CM, Lamaze C (2012) Membrane trafficking and signaling: two sides of the same coin. Semin Cell Dev Biol 23: 154–164. 22085846 10.1016/j.semcdb.2011.11.002

